# Health Care Use and Spending Among Need-Based Subgroups of Medicare Beneficiaries With Full Medicaid Benefits

**DOI:** 10.1001/jamahealthforum.2023.0973

**Published:** 2023-05-12

**Authors:** Brystana G. Kaufman, Kelley A. Jones, Melissa A. Greiner, Abhigya Giri, Lucas Stewart, Amanda He, Amy G. Clark, Donald H. Taylor, M. Kate Bundorf, Rebecca G. Whitaker, Courtney H. Van Houtven, Aparna Higgins

**Affiliations:** 1 Duke Margolis Center for Health Policy, Duke University, Durham, North Carolina; 2 Population Health Sciences, Duke University School of Medicine, Durham, North Carolina; 3 Center of Innovation to Accelerate Discovery and Practice Transformation (ADAPT), Durham VA Medical Center, Durham, North Carolina; 4 Founder, Ananya Health Solutions LLC, Dunn Loring, Virginia; 5 Sanford School of Public Health Policy, Duke University, Durham, North Carolina

## Abstract

**Importance:**

Beneficiaries dual eligible for Medicare and Medicaid account for a disproportionate share of expenditures due to their complex care needs. Lack of coordination between payment programs creates misaligned incentives, resulting in higher costs, fragmented care, and poor health outcomes.

**Objective:**

To inform the design of integrated programs by describing the health care use and spending for need-based subgroups in North Carolina’s full benefit, dual-eligible population.

**Design, Setting, and Participants:**

This cross-sectional study using Medicare and North Carolina Medicaid 100% claims data (2014-2017) linked at the individual level included Medicare beneficiaries with full North Carolina Medicaid benefits. Data were analyzed between 2021 and 2022.

**Exposure:**

Need-based subgroups: community well, home- and community-based services (HCBS) users, nursing home (NH) residents, and intensive behavioral health (BH) users.

**Measures:**

Medicare and Medicaid utilization and spending per person-year (PPY).

**Results:**

The cohort (n = 333 240) comprised subgroups of community well (64.1%, n = 213 667), HCBS users (15.0%, n = 50 095), BH users (15.2%, n = 50 509), and NH residents (7.5%, n = 24 927). Overall, 61.1% reported female sex. The most common racial identities included Asian (1.8%), Black (36.1%), and White (58.7%). Combined spending for Medicare and Medicaid was $26 874 PPY, and the funding of care was split evenly between Medicare and Medicaid. Among need-based subgroups, combined spending was lowest among community well at $19 734 PPY with the lowest portion (38.5%) of spending contributed by Medicaid ($7605). Among NH residents, overall spending ($68 359) was highest, and the highest portion of spending contributed by Medicaid (70.1%). Key components of spending among HCBS users’ combined total of $40 069 PPY were clinician services on carrier claims ($14 523) and outpatient facility services ($9012).

**Conclusions and relevance:**

Federal and state policy makers and administrators are developing strategies to integrate Medicare- and Medicaid-funded health care services to provide better care to the people enrolled in both programs. Substantial use of both Medicare- and Medicaid-funded services was found across all need-based subgroups, and the services contributing a high proportion of the total spending differed across subgroups. The diversity of health care use suggests a tailored approach to integration strategies with comprehensive set benefits that comprises Medicare and Medicaid services, including long-term services and supports, BH, palliative care, and social services.

## Key Points

**Question:**

For Medicare beneficiaries with full Medicaid benefits, how does the use of Medicare and Medicaid services and spending by payer differ across need-based subgroups?

**Findings:**

This cross-sectional study including 333 240 Medicare and Medicaid beneficiaries found that substantial use of Medicare- and Medicaid-funded services across all subgroups and describes the services contributing the most to total spending differed across subgroups. Medicaid-funded services, including community-based services and nursing home care, were key contributors to total spending among subgroups needing long-term care.

**Meaning:**

The diversity of health care use among dual-eligible beneficiaries requires integration strategies with comprehensive combined Medicare-Medicaid benefits to support whole-person, beneficiary-centered care for dual-eligible beneficiaries and their families.

## Teaser

This cross-sectional study examines how the use of Medicare and Medicaid services and spending by payer differ across need-based subgroups.

## Introduction

Medicare and Medicaid fund different benefits and services, and integration of the 2 programs is critical to better serve the 12 million people enrolled in both programs.^1^ Dual-eligible beneficiaries are, on average, sicker and frailer than other Medicare- or Medicaid-only beneficiaries because many are living with multiple chronic comorbidities and experience functional limitations, cognitive impairments, and mental health conditions.^2^ The complex care needs of dual-eligible beneficiaries account for a disproportionate share of expenditures in both Medicare and Medicaid.^3^ Lack of coordination between programs creates misaligned incentives for payers and physicians, resulting in higher costs, fragmented care, and poor health outcomes.^4^

Although evidence is limited, integrated models are intended to help align financial incentives with overall beneficiary experience and outcomes across the care continuum.^5,6^ Yet, relatively few dual-eligible beneficiaries are enrolled in integrated programs, such as the Program for All-Inclusive Care for the Elderly (PACE), or Medicare-Medicaid–managed care plans including the Financial Alignment Initiative (FAI), and Dual Special Needs Plans (D-SNPs). The PACE provides fully integrated financial, health care, and administrative processes; however, PACE models are challenging to scale. Overall, D-SNPs are more widely used than PACE, with 3.8 million dual-eligible beneficiaries enrolled nationwide and varying degrees of coordination and integration between Medicare and Medicaid across states. Despite Centers for Medicare & Medicaid Services (CMS) and state efforts, only around 10% of dual-eligible beneficiaries nationally are enrolled in programs that integrate Medicare and Medicaid care models, payments, and administrative processes.^7^

Population heterogeneity poses challenges in expanding access to, and enrollment in, integrated payment models. Beneficiaries enroll in Medicare based on age or long-term disability, and qualify for state Medicaid based on income or health care needs. Disabled adults comprise more than half of the dual-eligible population but only 15% of the general Medicare population.^8^ In addition to physical medical care, dually eligible beneficiaries often need behavioral health (BH) services, long-term services and supports (LTSS), and social supports.^3^ Long-term services and supports include both home and community-based services (HCBS) and facility-based care. Evaluations informing Medicare-Medicaid integration must consider average utilization and cost patterns of dual-enrollees across both programs, including among beneficiary subgroups with different health care needs.

Many studies of health outcomes use only Medicare spending and outcomes for this population, with few incorporating Medicaid data as well.^9,10,11,12^ The Medicare Payment Advisory Commission (MedPAC) has recently provided high-level data for the whole population,^8^ but did not examine cost and use variations across key subgroups, such as BH users or HCBS waiver participants. Other studies with linked Medicare-Medicaid claims focused on a specific type of service, subpopulation, or cost bracket.^13,14,15^ However, services with the highest spending may vary for different subgroups due to diverse care needs and differing access to services through Medicaid waiver programs.

Evaluating Medicaid spending is challenging in states with high use of Medicaid-managed care organizations (MCO). Encounter data for MCOs are less accurate compared with fee-for-service (FFS) claims data, which are used for reimbursement.^16,17^ In North Carolina (NC), however, more than 95% of dual-eligible beneficiaries were served by FFS Medicaid in 2019. Thus, analysis of the NC dual-eligible FFS population is more representative of the diversity of dual enrollees than in states where managed care is more prevalent. Compared with states with high managed care, evaluation of spending in NC may be more generalizable and applicable for policy recommendations due to availability of claims data on most of the Medicaid population. The NC population is also diverse in race and rurality and provides relevant context for other diverse states.^18,19,20^

Federal and state policy makers and administrators are currently developing strategies for Medicare-Medicaid integration.^21^ To inform the design of integrated programs that will meet the diverse needs of dual enrollees, we describe the Medicare and Medicaid health care use and spending for subgroups with different health care needs in NC. The state of NC is currently implementing Medicaid transformation from FFS to Medicaid value-based care models. We examined FFS spending both overall and for need-based subgroups defined from the Medicaid program perspective to support federal and state decision-making.

## Methods

This cross-sectional study used linked Medicare and Medicaid claims for 100% of beneficiaries dually enrolled in NC Medicaid and Medicare for at least 1 month (16 days within a calendar month) between January 1, 2014, and December 31, 2017 (eFigure 1 in Supplement 1). Utilization and spending were evaluated among a subcohort of beneficiaries with at least 1 month of enrollment in Medicare FFS programs A and B and no enrollment in Medicare Advantage. Medicaid in NC was primarily FFS during the study period. The PACE enrollees were included in the initial cohort we used to evaluate enrollment in capitated models like PACE and Medicare Advantage; however, beneficiaries with PACE enrollment during the study period were excluded from the evaluation of cost and use due to incomplete information on health care use. The Duke Health institutional review board and North Carolina Department of Health and Human Services reviewed and approved this study. This report followed Strengthening the Reporting of Observational Studies in Epidemiology (STROBE) reporting guidelines. The Duke Health institutional review board approved a waiver of informed consent and Health Insurance Portability and Accountability Act of 1996 (HIPAA) authorization for this secondary analysis.

### Need-Based Subgroups

We defined 4 need-based subgroups. First, community well included beneficiaries who did not meet criteria for any of the other need-based subgroups or 1915(c) or 1915(b) waiver enrollment (eFigure 1 in Supplement 1); however, community well might include high-need individuals with barriers to accessing care or receiving facility care for fewer than 100 days. Second, HCBS users included beneficiaries with any HCBS claims, specifically defined as personal care services, to identify individuals receiving long-term care in their home, assisted living or adult day home. Third, the nursing home group included beneficiaries with 100 or more consecutive days in a skilled nursing facility, identified with Medicaid place of service code indicating skilled nursing facility, nursing facility, or custodial care facility (regardless of Medicare payment status) in alignment with NC statute. Fourth, the high-intensity BH services users included individuals with claims indicating serious mental illness, substance use disorder, or intellectual or developmental disability (IDD)^22^ in the study period. Other than community well, the need-based subgroups are not mutually exclusive; beneficiaries could qualify for multiple groups over the time period. Characteristics and definitions for 1915(b)(c) waiver enrollees are presented in eTables 2-4 in Supplement 1).

### Demographic Information

Patient demographics were extracted from the Medicaid member files, and Medicare values were used where Medicaid data were missing. Race and ethnicity data were self-reported in NC Medicaid and Medicare, and we retained missing values when unavailable in both data sources due to poor validity of imputation methods.^23^ Beneficiaries’ county of residence was classified as rural or urban based on NC Department of Health and Human Services guidelines.^24^ The CMS Chronic Conditions Warehouse (CCW) indicators identified those with a condition date prior to or during the study period.

### Utilization and Spending Measures

Utilization and spending outcomes were ascertained for months in which beneficiaries met the criteria for a full dual enrollee and were enrolled in Medicare FFS parts A and B and not Medicare Advantage (eTable 1 in the Supplement 1). We measured utilization separately for each payer, and measures were not summed to avoid overcounting services paid by both payers. For example, Medicare is the primary payer for most acute and postacute services, and Medicaid typically contributes to acute and postacute spending by reimbursing clinicians for Medicare copays and coinsurance, or additional days beyond Medicare limits. We were unable to distinguish short-term from long-term postacute services in the Medicaid claims because the Medicaid billing codes do not differ between the 2 types of services.

For spending, we summed Medicaid- and Medicare-financed spending for a given person. Spending was calculated as the total amount paid by Medicaid and Medicare, with a per-diem adjustment included in inpatient spending for Medicare. Medicaid spending measures included all Medicaid FFS payments including professional, facility, and dental claims. Medicare spending included all parts A and B claims. Payments were adjusted for inflation using the medical care component of the Consumer Price Index (CPI) to calculate all spending in 2017 dollars.

### Statistical Analysis

Descriptive characteristics for the overall cohort and by subgroup are reported using frequencies and proportions for categorical variables and median and interquartile ranges (IQRs) for continuous variables. Yearly utilization and spending amounts were calculated as the sum of events, services, days, or spending divided by the total person-years eligible for the utilization outcomes, with accompanying 95% confidence intervals. No statistical testing was conducted for this descriptive analysis. We used SAS (version 9.4; SAS Institute, Inc) for all analyses. The analyses were conducted between 2021 and 2022.

## Results

### Dual Enrollment

Our cohort included 333 240 NC Medicaid beneficiaries with full Medicaid benefits ever enrolled in Medicare during the study period. The median (IQR) age was 65 (IQR, 52-76) years, and 61.1% reported female identity. The most common racial identities included White (58.7%), Black (36.1%), and Asian (1.8%) (Table 1). Most beneficiaries maintained their full dual status throughout the entire study period or until death (71.4%); however, about 1 in 3 dual-eligible beneficiaries who were full-benefit dual-eligible (FBDE) at the start of the period lost their full-dual eligibility status, primarily via loss of Medicaid benefits (eTable 3 and eFigure 2 in Supplement 1).

**Table 1.** Characteristics of Need-Based Subgroups Among North Carolina (NC) Full-Benefit Dual-Eligible Beneficiaries, 2014 to 2017^a^^,^^b^

Variable	Overall	Community well	HCBS user	Nursing home resident	Intensive BH service user
No.	333 240	213 667	50 095	24 927	50 509
Demographics					
Age, median (IQR), y	65.0 (52.0-76.0)	64.0 (53.0-73.0)	70.0 (60.0-81.0)	82.0 (73.0-88.0)	50.0 (36.0-61.0)
Aged adult (≥65 y)	167 537 (50.3)	104 098 (48.7)	32 172 (64.2)	22 697 (91.1)	8831 (17.5)
Race and ethnicity					
American Indian, Alaska Native, Native Hawaiian, or Pacific Islander	4486 (1.3)	2957 (1.4)	868 (1.7)	138 (0.6)	528 (1.0)
Asian	6151 (1.8)	5428 (2.5)	344 (0.7)	53 (0.2)	276 (0.5)
Black	120 197 (36.1)	73 419 (34.4)	24 699 (49.3)	5968 (23.9)	18 806 (37.2)
Multiracial or unknown	6904 (2.1)	4759 (2.2)	916 (1.8)	168 (0.7)	1194 (2.4)
White	195 502 (58.7)	127 104 (59.5)	23 268 (46.4)	18 600 (74.6)	29 705 (58.8)
Hispanic ethnicity	11 715 (3.5)	9740 (4.6)	806 (1.6)	258 (1.0)	942 (1.9%)
Sex					
Female	203 534 (61.1)	128 830 (60.3)	33 639 (67.2)	17 640 (70.8)	25 148 (49.8)
Male	129 706 (38.9)	84 837 (39.7)	16 456 (32.8)	7287 (29.2)	25 361 (50.2)
Rural residence	104 276 (31.3)	65 651 (30.7)	17 689 (35.3)	7086 (28.4)	14 458 (28.6)
Chronic conditions count, median (IQR)	7.0 (3.0-11.0)	6.0 (3.0-10.0)	10.0 (7.0- 13.0)	11.0 (8.0-13.0)	5.0 (2.0-9.0)
Died during study period^c^	47 240 (22.8)	22 740 (18.8)	12 117 (30.7)	7570 (55.4)	4191 (11.3)

Abbreviations: HCBS, home and community-based services; BH, behavioral health.

^a^ Unless otherwised noted, data are reported as number (percentage) of beneficiaries.^.^Includes NC Medicaid beneficiaries with full Medicaid benefits ever enrolled in Medicare during the study period. Beneficiaries may belong to 1 or more need-based subgroups within the study period.

^b^ 1915 waiver populations results presented in the eTable 2 in Supplement 1.

^c^ Mortality among beneficiaries who were full benefit dual-eligible in January 2014 (n = 206 874).

### Need-Based Subgroup Profiles

Dual-eligible beneficiaries could be included in multiple need-based subgroups over the 4-year period; see eTable 4 in Supplement 1 for overlap between subgroups. Most of the cohort, 64.1% (n = 213 667), were included in the community well subgroup, which included people who did not meet criteria for the other need-based subgroups at any point in the period. The HCBS subgroup included 15.0% (n = 50 095), BH subgroup included 15.2% (n = 50 509), and the nursing home subgroup included 7.5% (n = 24 927).

The need-based subgroups differed in their demographics and health status (Table 1) as well as program enrollment in Medicare Parts A, B, C, and D (Table 2). Among community well, 34.4% were Black, 30.7% had a rural residence, and 48.7% were older than 65 years. Community well subgroup members had complex care needs, with 54.8% of beneficiaries reporting 6 or more chronic conditions (median of 6 chronic conditions), yet this group used fewer and less intensive health care resources compared with other subgroups. Among community well, 18.8% died during the 4-year study period. Overall, PACE enrollment was highest among the community well group at 1.2%. Enrollment in Part D was nearly 100% across all 5 subgroups; however, Part C or Medicare Advantage enrollment was more common among nursing home residents (44%) than other groups.

**Table 2.** Medicare Program Enrollment by Need-Based Subgroups, North Carolina (NC), 2014 to 2017^a^^,^^b^

Variable	Overall	Community well	HCBS user	Nursing home resident	Intensive BH service user
No.	333 240	213 667	50 095	24 927	50 509
Medicare benefit plans, any enrollment during the study period					
PACE	3150 (0.9)	2656 (1.2)	315 (0.6)	85 (0.3)	98 (0.2)
FFS hospital inpatient care (part A)^c^	289 840 (87.0)	184 709 (86.4)	44 217 (88.3)	18 585 (74.6)	47 558 (94.2)
FFS medical outpatient services (part B)^c^	291 789 (87.6)	185 997 (87.0)	44 738 (89.3)	18 604 (74.6)	47 840 (94.7)
Medicare Advantage (part C)	82 158 (24.7)	53 030 (24.8)	11 836 (23.6)	11 038 (44.3)	8412 (16.7)
Prescription drug coverage (part D)	328 419 (98.6)	209 771 (98.2)	49 929 (99.7)	24 530 (98.4)	50 132 (99.3)

Abbreviations: HCBS, home and community-based services; BH, behavioral health; PACE, Program of All-Inclusive Care for the Elderly; FFS, fee-for-service.

^a^ Unless otherwised noted, data are reported as number (percentage) of beneficiaries. Includes NC Medicaid beneficiaries with full Medicaid benefits ever enrolled in Medicare during the study period. Beneficiaries may belong to 1 or more need-based subgroups within the study period. The values represent beneficiaries with any enrollment (≥1 month) in each benefit plan during the study period, out of all beneficiaries in that subgroup. Beneficiaries may switch plans over time, resulting in Parts A/B and Part C not summing to 100%.

^b^ 1915 Waiver populations results presented in eTables 2 and 3 Supplement 1.

^c^ Parts A and B were counted in the months where they enrolled in each program, respectively, but not enrolled in Plan C (ie, Parts A and B while FFS).

Compared with community well, HCBS users had higher prevalence of Black and rural beneficiaries and poorer health status, with a higher median number of chronic conditions (10), and higher mortality during the study period (30.7%). Compared with community well, the BH users had lower prevalence of beneficiaries older than 65 years (17.5%), higher prevalence of Black (37.2%) beneficiaries, and fewer chronic conditions. Compared with the community well, the nursing home subgroup had higher prevalence of beneficiaries older than 65 years (91.1%) and lower prevalence of Black beneficiaries (23.9%). The nursing home subgroup was the most likely to have 6 or more chronic conditions (88.7%), with a median of 11 chronic conditions, and experienced the highest mortality (55.4%) of all need-based subgroups.

### Service Utilization by Need-Based Subgroup

Because Medicare is the primary payer and Medicaid covers beneficiary copays and non-Medicare covered days, we expected both Medicare and Medicaid claims would exist for acute care events. Our linked data showed that fewer acute care events, such as ED visit, hospital admission, and inpatient days, were observed in Medicaid than Medicare (Table 3). The difference between acute events observed in Medicare and events observed in Medicaid claims was highest among the nursing home resident subgroup.

**Table 3.** Health Care Utilization Rates per Person-Year^a^ by Need-Based Subgroups

Variable	Overall (95% CI)	Community well (95% CI)	HCBS user (95% CI)	Nursing home resident (95% CI)	Intensive BH service user (95% CI)
**Emergency department visits**					
Medicare	1.53 (1.53-1.53)	1.22 (1.22-1.23)	2.17 (2.16-2.18)	1.12 (1.11-1.13)	2.17 (2.16-2.18)
Medicaid	1.25 (1.25-1.25)	1.02 (1.02-1.02)	1.64 (1.64-1.65)	0.51 (0.50-0.51)	1.96 (1.95-1.97)
**Hospital admissions**					
Medicare	0.48 (0.48-0.48)	0.39 (0.38-0.39)	0.73 (0.73-0.74)	0.54 (0.53-0.54)	0.54 (0.53-0.54)
Medicaid	0.28 (0.28-0.28)	0.24 (0.23-0.24)	0.45 (0.45-0.45)	0.24 (0.23-0.24)	0.28 (0.27-0.28)
**Inpatient days**					
Medicare	3.72 (3.72-3.73)	2.76 (2.75-2.77)	5.81 (5.80-5.83)	4.24 (4.22-4.26)	5.30 (5.29-5.31)
Medicaid	2.20 (2.20-2.21)	1.55 (1.54-1.55)	3.17 (3.16-3.18)	1.89 (1.87-1.90)	3.59 (3.58-3.60)
Post-acute SNF days^b^					
Medicare	7.04 (7.03-7.04)	4.86 (4.85-4.86)	7.59 (7.57-7.61)	42.01 (41.95-42.08)	4.26 (4.25-4.27)
**Home health days**					
Medicare	10.38 (10.38-10.39)	4.96 (4.96-4.97)	30.35 (30.31-30.38)	4.43 (4.40-4.45)	10.80 (10.79-10.82)
Medicaid	2.84 (2.83-2.84)	0.76 (0.75-0.76)	7.12 (7.10-7.13)	0.90 (0.89-0.91)	3.08 (3.07-3.09)
**Hospice days**					
Medicare	3.50 (3.50-3.51)	2.49 (2.49-2.50)	6.61 (6.60-6.63)	9.12 (9.09-9.15)	1.43 (1.42-1.43)
Medicaid	1.65 (1.65-1.66)	1.59 (1.59-1.60)	0.94 (0.94-0.95)	8.27 (8.24-8.29)	0.60 (0.59-0.60)
**Total BH services**					
Medicare	2.34 (2.34-2.35)	1.25 (1.25-1.26)	3.40 (3.39-3.41)	2.90 (2.88-2.92)	5.96 (5.95-5.97)
Medicaid	10.42 (10.41-10.42)	0.80 (0.80-0.81)	6.07 (6.06-6.08)	1.67 (1.66-1.68)	38.57 (38.53-38.60)
Medicaid-only services					
SNF/nursing home days	35.21 (35.19-35.22)	20.78 (20.76-20.79)	21.02 (20.99-21.04)	315.45 (315.28-315.63)	21.16 (21.13-21.18)
Intermediate care facility days	1.78 (1.77-1.78)	0 (0-0)	0.13 (0.13-0.14)	0.17 (0.16-0.17)	8.95 (8.93-8.96)
Supplemental services days^c^	10.87 (10.86-10.88)	0.49 (0.49-0.49)	6.61 (6.59-6.62)	1.57 (1.56-1.59)	52.40 (52.36-52.44)
HCBS days	37.27 (37.25-37.28)	0 (0-0)	209.74 (209.66-209.82)	11.34 (11.30-11.37)	47.73 (47.69-47.76)

Abbreviations: HCBS, home and community based services; SNF, skilled nursing facility; BH, behavioral health.

^a^ 283 322 Patients with at least 1 month of full-benefit dual eligibility plus Medicare fee-for-service included in the analysis. Rates shown as utilization per person-year.

^b^ Services covered by Medicare only.

^c^ Measured as days with supplemental services under the 1915(b) waiver benefits provided through the local management entities including personal care services and supports for individuals with intellectual and developmental disability or BH needs.

For nursing facility use, the Medicare and Medicaid days often reflect different types of care. Medicaid nursing facility days are primarily long-term care, but also include Medicaid payments for post-acute care days that overlap with Medicare post-acute days. Medicare-covered skilled nursing facility days are most common among nursing home residents. Other utilization measures included home health days, hospice days, and total BH service visits. More home health visits and hospice days were observed in Medicare than Medicaid claims. More behavioral health service visits were observed in Medicaid than Medicare claims.

### Medicaid and Medicare Spending by Need-Based Subgroup

In the utilization subcohort, overall, combined total spending for Medicare and Medicaid was $26 874 PPY (Figure). In the overall dual population, total spending PPY was evenly distributed between Medicare ($14 175) and Medicaid ($12 698). The services contributing the most to combined spending were outpatient facility care ($7138) and professional/carrier claims ($6214). Among outpatient facility spending, a greater portion was funded by Medicaid ($4199) than Medicare ($2939). The next substantial contributor was postacute and long-term care spending at $4731 for Medicaid and $1341 for Medicare spending PPY. Inpatient services represented less than 20% of combined spending PPY at $5231 for Medicare and $86 for Medicaid (reflecting Medicare cost-sharing).

**Figure.**
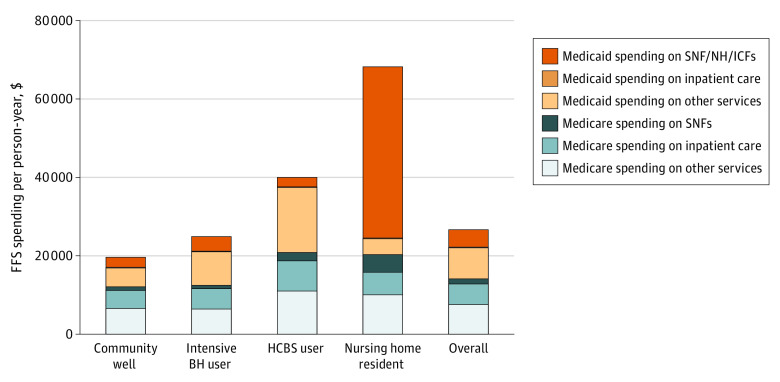
Decomposition of Overall Fee-for-Service (FFS) Medicaid and Medicare Spending per Person-Year by Need-Based Subgroup North Carolina (NC) dual-eligible Medicare and Medicaid beneficiaries, 2014 to 2017, were identified from the Medicare 100% sample inpatient, skilled nursing facility, home health, outpatient, carrier, durable medical equipment, and hospice claims linked to NC Medicaid equivalent claims at the beneficiary level. The sample includes 283 322 patients with at least 1 month of dual-eligibility for full NC Medicaid benefits and Medicare FFS included in the analysis. Rates shown as spending per person-year. Skilled nursing facilities, nursing homes, and intermediate care facilities (SNF/NH/ICFs) represent the institutional spending for these locations. Medicaid inpatient spending is depicted in yellow but has low values compared with the other categories (range, $17.73-$169.11 per person-year). BH indicates behavioral health; HCBS, home and community-based services.

Among the need-based subgroups, combined spending PPY was lowest among community well at $19 734 and the lowest portion (38.5%) of spending contributed by Medicaid ($7605). Among nursing home residents, overall spending ($68 359) was highest and the highest portion of spending by Medicaid (70.1%), predominately attributed to Medicaid spending on postacute and long-term care ($43 686) PPY. The HCBS subgroup had overall spending of $40 069 PPY and Medicaid accounted for 47.7% ($19 107) with the largest contributor to combined spending being professional/carrier claims ($14 523) and outpatient claims ($9012). Health care spending PPY is shown in eTables 5 to 10 in Supplement 1.

## Discussion

We found substantial use of both Medicare- and Medicaid-funded services across all need-based subgroups, demonstrating the need for both Medicare and Medicaid claims to build an accurate picture of dual-eligible beneficiaries’ needs to support care coordination, program evaluation, and policy administration. We also found that the proportion of total spending paid by Medicaid varied by need-based subgroup. Medicaid contributed up to 70% of total spending for high-need populations including nursing home residents. Overall, however, the financial burden of care for dual-enrollees was split evenly between Medicare and Medicaid. Our estimates for total spending among the dual-eligible population in NC was comparable to national estimates using similar data.^9^ These findings demonstrate the diverse health care needs within the dual populations, which has implications for state and federal policies considering strategies for Medicare-Medicaid integration.

States, supported by CMS, have been implementing innovative payment and delivery models to address the shortcomings of the current system and advance value-based care for dual-eligible beneficiaries. Three notable models include the PACE, FAI, and D-SNPs. The PACE model provides full financial integration in addition to integrated care and administrative processes for individuals aged 55 years and older who require nursing facility level of care. The FAI model mostly tested a capitated model whereby states, CMS, and plans entered into a contract, called a Medicare-Medicaid Plan (MMP), for a blended capitation rate. Although CMS has closed the window for new enrollment in the Financial Alignment Initiative demonstrations, D-SNPs have permanent authorization under the Bipartisan Budget Act of 2018. These D-SNPs are required to contract with the states (via State Medicaid Agency Contracts, SMACs) in which they operate, in addition to contracts with CMS, and must adhere to state and CMS requirements. Many states with D-SNPs have leveraged these SMACs as the primary vehicle for providing integrated services for full-benefit dual-eligible beneficiaries. For example, states can require D-SNP contracts in their state to meet CMS criteria for fully-integrated D-SNPs (FIDE-SNP).

The implications of this research for state and federal policies are particularly timely given the potential legislation pending at the national level to expand access to Medicare-Medicaid integrated programs. To increase access to integrated care, policy makers should consider expansion of PACE to new regions and populations. Multiple Senate and House bills (H.R.6770, S.1162, S.3626)^21^ propose expanding eligibility and geographic expansion for PACE, and other bills support integration planning at the state level (S.4264, S.4273, S.3630). Expansion of PACE can play an important role in future integration efforts in 2 ways. First, it allows more individuals who are eligible for PACE to enroll and receive integrated care. Second, PACE expansion increases opportunities for collaborative and innovative partnerships between non-PACE and PACE organizations serving dual-eligible beneficiaries. For example, in Massachusetts, managed care plans have partnered with PACE organizations to help deliver a PACE-like model in the community. In the absence of new legislation, states can use a combination of PACE and FIDE-SNPs to expand integrated options for dual-eligible beneficiaries.

Given the overlap and churn between need-based groups, a single plan and program offering the broad range of services will minimize disruption to care during transitions where integration and continuity are most needed. Integrated plans should include comprehensive Medicare and Medicaid services, including LTSS, BH, social services and other supplemental benefits to support whole-person care. Medicare benefit packages have historically been restricted; however, more flexible benefits became available with the introduction of Medicare Advantage special needs plans in 2006, and have expanded over time to include LTSS, BH, palliative care, and other support services. New regulations require FIDE-SNPs to provide comprehensive Medicare and Medicaid services, including either long-stay nursing home care or behavioral health services. States contracting with D-SNPs should ensure options for integrated D-SNPs with comprehensive benefits are available in all regions, including rural areas.

Similar to PACE, integrated programs should conduct integrated assessments of dual-eligible beneficiaries’ health risks, needs, and preferences to provide a comprehensive understanding of the whole person. A standard functional assessment tool would ensure development of an individualized person-centered care plan that is designed to meet the unique needs of high-risk beneficiaries and should be updated as needed to address beneficiaries’ needs as they change over time and across care settings. Use of combined Medicare-Medicaid data can support implementation of tailored approaches for integration and care coordination to best serve each subgroup. Not only do Medicare and Medicaid fund different services, creating blind-spots in care, there are gaps in claims for services that typically appear in both sources of data. The difference between acute events observed in Medicare and events observed in Medicaid claims was highest among the nursing home resident subgroup, indicating that the need for Medicare claims data to facilitate care coordination is greatest in this group.

The business case for investing in preventive care and Medicaid services is strengthened when Medicare and Medicaid plans are financially aligned, meaning the same entity provides both Medicare and Medicaid services. In the context of financial alignment, improvements in LTSS (funded by Medicaid) that prevent acute care use (funded by Medicare) accrue to the same entity, creating the incentive for prevention of Medicare costs for Medicaid plans. In Medicare-only populations, inpatient spending accounts for the largest portion of total spending. This cross-sectional study found that acute and postacute services were not contributing the most to combined Medicare-Medicaid spending for some subgroups of dual-eligible beneficiaries. This evidence points to shifting facility-based services to home and community-based settings, with appropriate oversight to ensure high-quality care, as a potential for improving efficiency in resource use and value of care.^9,14,15,25^

### Limitations

These results may not be representative of dual enrollees in states other than NC. Other states’ prevalence of subgroups, FFS Medicaid, and waiver participation might differ from NC because state programs and coverage policies vary. States frequently offer multiple different Medicaid waivers and care models to meet the needs of different groups; for example, NC has 1115^26^ and 1915(b) and (c) waivers for integrated mental health, HCBS, and people with IDD.^26^

## Conclusions

This cross-sectional study found that needs and service use differed by subgroups, and the types of services contributing to spending also differed from evidence in Medicare-only populations. The diversity of health care use suggests that a tailored approach to integration strategies with comprehensive set benefits that comprises Medicare and Medicaid services, including LTSS, BH, palliative care, and social services is needed. These findings may inform the design of integrated programs that could improve access to whole-person, beneficiary-centered care for dual-eligible beneficiaries and their families.

## Notes

Supplement 1. eFigure 1. Medicare-Medicaid linkage and cohort inclusion criteria eTable 1. Utilization and cost variable algorithms eTable 2. Characteristics of need-based subgroups among NC full-benefit dual-eligible beneficiaries, 2014-2017 eTable 3. Dual status in January 2014 by need-based subgroup eFigure 2. Changes in Dual Status Throughout the Study Period by Need-Based Subgroup, among those who were full-benefit dual eligible (FBDE) beneficiaries in January 2014 eTable 4. Overlap in Beneficiaries Between Need-Based Subgroups eTable 5. Proportion of total fee-for-service healthcare spending per person-year funded by Medicaid Programs among North Carolina full benefit dual eligible, overall eTable 6. Proportion of total fee-for-service healthcare spending per person-year funded by Medicaid Programs among Community Well beneficiaries eTable 7. Proportion of total fee-for-service healthcare spending per person-year funded by Medicaid Programs among 1915(c) Waiver beneficiaries eTable 8. Proportion of total fee-for-service healthcare spending per person-year funded by Medicaid Programs among Home- and Community-Based Service (HCBS) users eTable 9. Proportion of total fee-for-service healthcare spending per person-year funded by Medicaid Programs among Intensive Behavioral Health service users eTable 10. Proportion of total fee-for-service healthcare spending per person-year funded by Medicaid Programs among Nursing Home Residents

Supplement 2. Data Sharing Statement
