# Supplementary material for: Health Care Use and Spending Among Need-Based Subgroups of Medicare Beneficiaries With Full Medicaid Benefits
Source: JAMA Health Forum. 2023 May 12;4(5):e230973. doi: 10.1001/jamahealthforum.2023.0973 (PMC10182424; doi:10.1001/jamahealthforum.2023.0973)
Supplement: Supplement 1. — eFigure 1. Medicare-Medicaid linkage and cohort inclusion criteria eTable 1. Utilization and cost variable algorithms eTable 2. Characteristics of need-based subgroups among NC full-benefit dual-eligible beneficiaries, 2014-2017 eTable 3. Dual status in January 2014 by need-based subgroup eFigure 2. Changes in Dual Status Throughout the Study Period by Need-Based Subgroup, among those who were full-benefit dual eligible (FBDE) beneficiaries in January 2014 eTable 4. Overlap in Beneficiaries Between Need-Based Subgroups eTable 5. Proportion of total fee-for-service healthcare spending per person-year funded by Medicaid Programs among North Carolina full benefit dual eligible, overall eTable 6. Proportion of total fee-for-service healthcare spending per person-year funded by Medicaid Programs among Community Well beneficiaries eTable 7. Proportion of total fee-for-service healthcare spending per person-year funded by Medicaid Programs among 1915(c) Waiver beneficiaries eTable 8. Proportion of total fee-for-service healthcare spending per person-year funded by Medicaid Programs among Home- and Community-Based Service (HCBS) users eTable 9. Proportion of total fee-for-service healthcare spending per person-year funded by Medicaid Programs among Intensive Behavioral Health service users eTable 10. Proportion of total fee-for-service healthcare spending per person-year funded by Medicaid Programs among Nursing Home Residents [file jamahealthforum-e230973-s001.pdf]

Supplemental Online Content

Kaufman BG, Jones KA, Greiner MA, et al. Health care use and spending among need-based subgroups of Medicare beneficiaries with full Medicaid benefits. *JAMA Health Forum*. 2023;4(5):e230973. doi:10.1001/jamahealthforum.2023.0973

eFigure 1. Medicare-Medicaid linkage and cohort inclusion criteria
eTable 1. Utilization and cost variable algorithms
eTable 2. Characteristics of need-based subgroups among NC full-benefit dual-eligible beneficiaries, 2014-2017
eTable 3. Dual status in January 2014 by need-based subgroup
eFigure 2. Changes in Dual Status Throughout the Study Period by Need-Based Subgroup, among those who were full-benefit dual eligible (FBDE) beneficiaries in January 2014
eTable 4. Overlap in Beneficiaries Between Need-Based Subgroups
eTable 5. Proportion of total fee-for-service healthcare spending per person-year funded by Medicaid Programs among North Carolina full benefit dual eligible, overall
eTable 6. Proportion of total fee-for-service healthcare spending per person-year funded by Medicaid Programs among Community Well beneficiaries
eTable 7. Proportion of total fee-for-service healthcare spending per person-year funded by Medicaid Programs among 1915(c) Waiver beneficiaries
eTable 8. Proportion of total fee-for-service healthcare spending per person-year funded by Medicaid Programs among Home- and Community-Based Service (HCBS) users
eTable 9. Proportion of total fee-for-service healthcare spending per person-year funded by Medicaid Programs among Intensive Behavioral Health service users
eTable 10. Proportion of total fee-for-service healthcare spending per person-year funded by Medicaid Programs among Nursing Home Residents

This supplemental material has been provided by the authors to give readers additional information about their work.

© 2023 American Medical Association. All rights reserved.

eFigure 1. Medicare-Medicaid linkage and cohort inclusion criteria

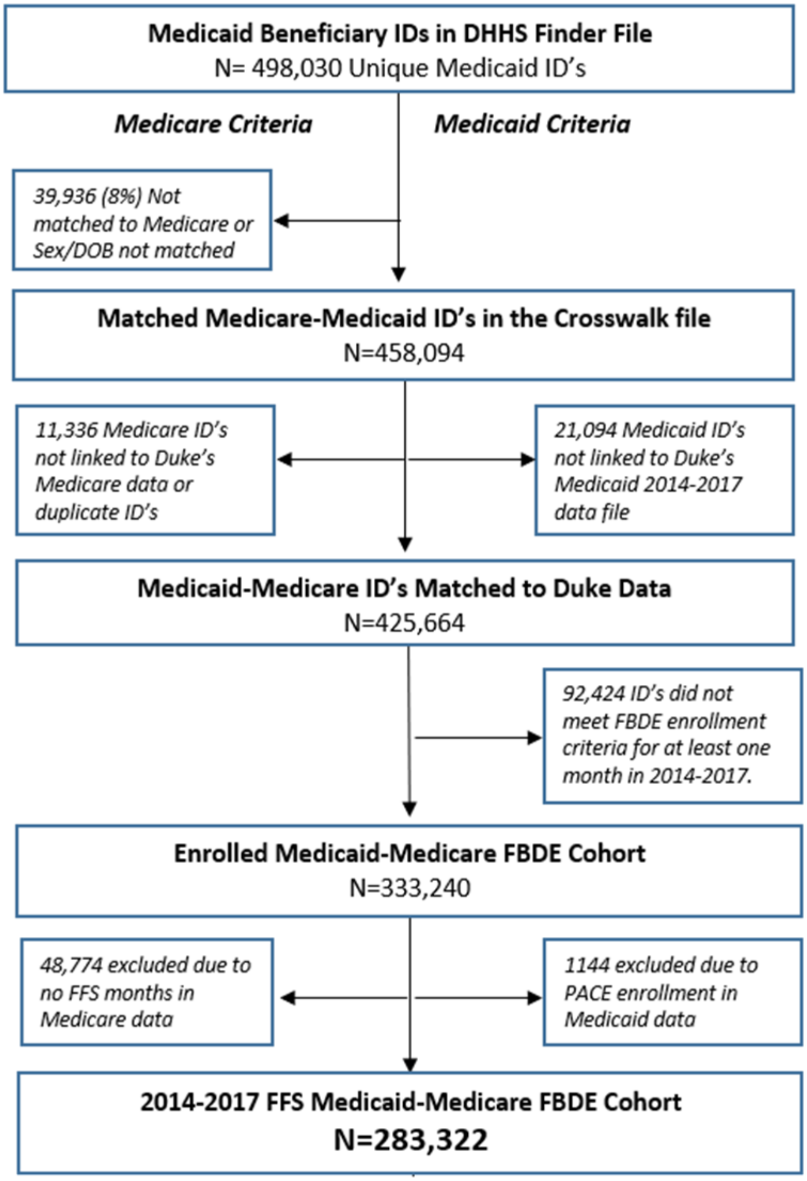

Source: Authors' analysis of data for 2014–17 from the 100% Linked Medicare-Medicaid Claims

© 2023 Kaufman B, et al, *JAMA Health Forum*

eTable 1. Utilization and cost variable algorithms

Characteristic	Definition	Utilization	Spending
Emergency department visit	Revenue code 0981 or 045*	Number of distinct all-cause ED visits per person	N/A
ED visit with admit	Revenue code 0981 or 045* plus the following:<br>Medicare: Revenue code 0981 or 045* on inpatient claim<br>Medicaid: At least one of the following:<br>• Patient status code: admitted to inpatient hospital, transferred to a DRG hospital, transferred to a cancer center/children's hospital, discharged to a federal hospital, or discharged to a critical access hospital<br>• Inpatient admission with a start date equal to the ED visit end date	Number of distinct all-cause ED visits with admission per person	N/A
ED visit without admit	Medicare: Revenue code 0981 or 045* on outpatient claim<br>Medicaid: Revenue code 0981 or 045* but does not meet the criteria for ED visit with admit.	Number of distinct all-cause ED visits with admission per person	N/A
Days in Inpatient Settings	Medicare: Any inpatient claim (Inpatient files)<br>Medicaid (must meet all requirements):<br>• Institutional claim<br>• Valid revenue code<br>• At least one of the following: place of service code in (21, 51, 56, 61); Valid DRG code; revenue code 100-219<br>• Place of service is not (31, 32, 54) and billing provider taxonomy is not 314000000X (nursing facility)	Total count of days January 1, 2014 – December 31, 2017 calculated using the service start and end dates (inclusive) and summing across all hospitalizations in the period.	Medicare: sum PMT_AMT + (PER_DIEM*UTIL_DAY) in the IP files<br>Medicaid: sum TTL_LNE_NET_AMT from qualifying claim lines
Hospital Admissions	Inpatient setting claims, identified above, excluding claims where the discharge status is 'still a patient'	Number of distinct all-cause hospital admissions per person	N/A
Days in Skilled Nursing Facilities - Medicare only	Medicare: use all claims in the SNF files<br>Medicaid: N/A	Total count of days between January 1, 2014 – December 31, 2017 calculated using the service start and end dates (inclusive) and summing across all SNF stays in the period.	Medicare: PMT_AMT in SNF files<br>Medicaid: N/A
Days in post-acute/longer term care – Medicaid only	Medicare: N/A<br>Medicaid: Combination of SNF/Nursing home and Intermediate Care Facility service days	Total count of days calculated by summing the following two categories	Medicare: N/A<br>Medicaid: Sum the following SNF/nursing home and intermediate care facility

© 2023 Kaufman B, et al, JAMA Health Forum

Characteristic	Definition	Utilization	Spending
			categories
Days in SNF/Nursing homes – Medicaid only	<u>Medicare</u>: N/A<br><u>Medicaid</u>: Institutional claims with billing provider taxonomy code = 314000000X (skilled nursing facility) or place of service codes skilled nursing facility or nursing facility	Total count of days between January 1, 2014 – December 31, 2017 calculated using the service start and end dates (inclusive) and summing across all SNF/NH stays in the period.	<u>Medicare</u>: N/A<br><u>Medicaid</u>: sum TTL_LNE_NET_AMT from qualifying claim lines
Days in intermediate care facilities – Medicaid only	<u>Medicare</u>: N/A<br><u>Medicaid</u>: Institutional claims place of service code for Intermediate Care Facility for Individuals with Intellectual Disabilities and billing provider taxonomy code is not 314000000X (skilled nursing facility)	Total count of days between January 1, 2014 – December 31, 2017 calculated using the service start and end dates (inclusive) and summing across all ICF stays in the period.	<u>Medicare</u>: N/A<br><u>Medicaid</u>: sum TTL_LNE_NET_AMT from qualifying claim lines
Long Term Services and Supports (LTSS) – Medicaid Only	<u>Medicare</u>: N/A<br><u>Medicaid</u>: Professional claims that meet all three of the following criteria:<br>• A claim for Personal Care Services (HDR_TYP_CD = '6')<br>• CPT code 99509 (home visit for assistance with activities of daily living and personal care)<br>• Procedure modifier code HA, HB, HC, HH, HI, HQ, SC, or TT)	Total count of days between January 1, 2014 – December 31, 2017 calculated using the service start and end dates (inclusive) and summing across all SNF/NH stays in the period.	<u>Medicare</u>: N/A<br><u>Medicaid</u>: sum TTL_LNE_NET_AMT from qualifying claim lines<br><br>*This value is a subset of the Carrier costs*
Behavioral Health Services days	Search all claims for the following:<br>• Psychiatric diagnostic evaluation: CPT codes 90791, 90792<br>• Psychotherapy: CPT codes 90832-90834, 90836-90840, 90846, 90847, 90849, 90853<br>• Evaluation and management (E&M) services with a psychologist or psychiatrist: CPT codes 99201-99205, 99211-99215, 99217-99223, 99231-99233, 99241-99245, 99251-99255, 99281-99288, 99304-99310, 99315-99316, 99318, 99324-99328, 99334-99337, 99341-99345, 99347-99350, 99366, 99401-9404, 99406-99409, 99411-99412 -and- psychology or psychiatrist taxonomy on the claim (beginning with 2084 or 103T)<br>• Neurostimulation services: CPT codes 90867-90870<br>• Psychiatric emergency department visit: Revenue code	Total number of BH service days are summed per subcategory (e.g., psychotherapy) calculated using the start and end dates (inclusive) and summing across that subcategory and then across all subcategories for the total number of BH service days.	<u>Medicare</u>: N/A<br><u>Medicaid</u>: sum TTL_LNE_NET_AMT from qualifying claim lines<br><br>*This value is a subset of the other care setting costs*

© 2023 Kaufman B, et al, JAMA Health Forum

Characteristic	Definition	Utilization	Spending
	0981 or 045* -and- at least one behavioral health diagnosis on the claim<br>• Intensive behavioral health services: CPT codes H0012, H0013, H0017, H0018, H0019, H0046, H2020, S5145, H2036, S9484, H0015, H0035, H2012, H2035, S9480, H2022, H2033, H0010, H0014, H0020, H0040, H2034 -or- state category of service 0021, 0047, 0017, 0041 -or- revenue code 100, 183, 911, 919<br>• Collaborative care: CPT codes 99492, 99493, 99494<br>• Waiver services: Enrollment in the waiver program at the time of service –and- at least one of the following CPT codes: 97532, A0090, B4150, B4152-B4155, B4157-B4162, E0700, G9003, G9004, H0045, H2010, H2011, H2015, H2016, H2025, S5102, S5110, S5111, S5125, S5135, S5150, S5161, S5165, S5170, T1004, T1005, T1015, T1016, T1019, T1020, T1999, T2013, T2014, T2020, T2021, T2025, T2027-T2029, T2033, T2034, T2038-T2041, T4535, T4539, T5999,<br>• Applied behavior analysis: CPT codes 0359T, 0360T, 0361T, 0362T, 0363T, 0364T, 0365T, 0373T, 0374T, 0368T, 0369T, 0366T, 0367T, 0372T, 0370T, 0371T		
LME-MCO Services – Medicaid Only	Medicare: N/A<br>Medicaid: Institutional or Professional claim with the claim type identified as an encounter (managed care claim; clm_btch_doc_typ_cd='E') during a month in which the beneficiary is enrolled in the LME-MCO. Enrollment is based on a capitated payment made in that month for that beneficiary.	Total count of days between January 1, 2014 – December 31, 2017 calculated using the service start and end dates (inclusive) and summing across all LME-MCO services in the period.	Medicare: N/A<br>Medicaid: sum TTL_LNE_NET_AMT from qualifying claim lines<br><br>*This value is a subset of the other care setting costs*
Home Health days	Medicare: use all claims in the Medicare home health files<br>Medicaid:<br>• Institutional claim with at least one of the following: Revenue code 0023, 056*-060*; CPT code 99500-99602; claim=HH; type of bill=301-399; or place of service=13 (assisted living)<br>• AND patient does not have an OP facility visit that day (any of the following):<br>○ ED visit: revenue code 450-452, 456, 459 or CPT code 99281-99292, 99466-99476	Total count of days where the beneficiary received home health services between January 1, 2014 – December 31, 2017, calculated using the service start and end dates (inclusive) and summing across all home health services in the period.	Medicare: sum PMT_AMT in home health files<br>Medicaid: sum TTL_LNE_NET_AMT from qualifying claim lines

© 2023 Kaufman B, et al, JAMA Health Forum

Characteristic	Definition	Utilization	Spending
	○ Outpatient surgery: revenue code 360-362, 367, 369, 481, 490, 499, 790, 799 or CPT code 10021-36410, 36420-58999, 60000-69990, 92920-92944, 93501-93581, 0016T-0261T, 0392T-0393T, 0016U-0023U<br>○ Observation: revenue code 760- 762, 769 or CPT code 99217-99220<br>○ Ambulance: CPT code A0021-A0999<br>• Excludes DME claim lines (CPT codes A4206-A4650, A4661-A4926, A4933-A9999, E0100-E8002, K0001-K0902, L0100-L9999)		
Hospice days	Medicare: use all claims in the Medicare hospice files<br>Medicaid: place of service='34' (hospice)	Total count of days where the beneficiary received hospice services between January 1, 2014 – December 31, 2017, calculated using the service start and end dates (inclusive) and summing across all hospice services in the period.	Medicare: sum PMT_AMT in hospice files<br>Medicaid: sum TTL_LNE_NET_AMT from qualifying claim lines
Outpatient facility	Medicare: use all claims in the Medicare outpatient files<br>Medicaid: All Institutional claim lines that are not captured in the preceding outcome and spending categories	N/A	Medicare: sum PMT_AMT in outpatient files<br>Medicaid: sum TTL_LNE_NET_AMT from qualifying claim lines
Carrier	Medicare: use all claims in the Medicare carrier files<br>Medicaid: use all claims in the Professional (PR) files	N/A	Medicare: sum PMT_AMT in carrier files<br>Medicaid: sum TTL_NET_PAY_AMT in PR files, once per claim
DME	Medicare: use all claims in the Medicare DME files<br>Medicaid: CPT code A4206-A4650, A4661-A4926, A4933-A9999, E0100-E8002, K0001-K0902, or L0100-L9999 AND patient does not have an OP visit that day (see definition under Home Health outcome)	N/A	Medicare: sum PMT_AMT in DME files<br>Medicaid: sum TTL_LNE_NET_AMT from qualifying claim lines
Dental – Medicaid only	Medicare: N/A<br>Medicaid: use all claims in the Dental Medicaid files	N/A	Medicare: N/A<br>Medicaid: sum

© 2023 Kaufman B, et al, JAMA Health Forum

Characteristic	Definition	Utilization	Spending
			TTL_NET_PAY_AMT in Dental files, once per claim

© 2023 Kaufman B, et al, JAMA Health Forum

eTable 2. Characteristics of need-based subgroups among NC full-benefit dual-eligible beneficiaries, 2014-2017[a]

Variable	Overall	Community well	HCBS	Nursing home resident	Intensive BH service user	1915(c) waiver[b]	1915(b) waiver[c]
N	333,240	213,667	50,095	24,927	50,509	17,215	4,277
<u>Demographics</u>							
Age (years), Median (Q1, Q3)	65.0 (52.0, 76.0)	64.0 (53.0, 73.0)	70.0 (60.0, 81.0)	82.0 (73.0, 88.0)	50.0 (36.0, 61.0)	66.0 (46.0, 80.0)	36.0 (27.0, 47.0)
Aged adult (65 years and over)	167,537 (50.3%)	104,098 (48.7%)	32,172 (64.2%)	22,697 (91.1%)	8,831 (17.5%)	9,047 (52.6%)	189 (4.4%)
Race							
American Indian, Alaskan Native, Native Hawaiian, or Pacific Islander	4,486 (1.3%)	2,957 (1.4%)	868 (1.7%)	138 (0.6%)	528 (1.0%)	322 (1.9%)	27 (0.6%)
Asian	6,151 (1.8%)	5,428 (2.5%)	344 (0.7%)	53 (0.2%)	276 (0.5%)	159 (0.9%)	36 (0.8%)
Black	120,197 (36.1%)	73,419 (34.4%)	24,699 (49.3%)	5,968 (23.9%)	18,806 (37.2%)	6,766 (39.3%)	1,247 (29.2%)
Multi-Racial or unknown	6,904 (2.1%)	4,759 (2.2%)	916 (1.8%)	168 (0.7%)	1,194 (2.4%)	287 (1.7%)	72 (1.7%)
White	195,502 (58.7%)	127,104 (59.5%)	23,268 (46.4%)	18,600 (74.6%)	29,705 (58.8%)	9,681 (56.2%)	2,895 (67.7%)
Hispanic ethnicity	11,715 (3.5%)	9,740 (4.6%)	806 (1.6%)	258 (1.0%)	942 (1.9%)	269 (1.6%)	54 (1.3%)

© 2023 Kaufman B, et al, JAMA Health Forum

Variable	Overall	Community well	HCBS	Nursing home resident	Intensive BH service user	1915(c) waiver[b]	1915(b) waiver[c]
Sex							
Female	203,534 (61.1%)	128,830 (60.3%)	33,639 (67.2%)	17,640 (70.8%)	25,148 (49.8%)	11,637 (67.6%)	1,667 (39.0%)
Male	129,706 (38.9%)	84,837 (39.7%)	16,456 (32.8%)	7,287 (29.2%)	25,361 (50.2%)	5,578 (32.4%)	2,610 (61.0%)
Rural residence[d]	104,276 (31.3%)	65,651 (30.7%)	<17,700 (<36%)	7,086 (28.4%)	14,458 (28.6%)	<7,000 (<41%)	1,165 (27.2%)
Chronic conditions count Median (Q1, Q3)	7.0 (3.0, 11.0)	6.0 (3.0, 10.0)	10.0 (7.0, 13.0)	11.0 (8.0, 13.0)	5.0 (2.0, 9.0)	9.0 (4.0, 12.0)	2.0 (1.0, 5.0)
Died during study period[e]	47,240 (22.8%)	22,740 (18.8%)	12,117 (30.7%)	7,570 (55.4%)	4,191 (11.3%)	4,229 (28.5%)	209 (6.0%)

[*] Value not shown due to cell size suppression requirements. Values with "<" are used to prevent back-calculation of suppressed values.

[a] Includes NC Medicaid beneficiaries with full Medicaid benefits ever enrolled in Medicare during the study period. Beneficiaries may belong to one or more need-based subgroups within the study period.

[b] Includes waiver groups for medically fragile children (CAP-C) and adults (CAP-DA) who are at risk for institutionalization.

[c] Includes Innovations waiver 1915(b) waiver primarily serving individuals with intellectual or developmental disabilities and Traumatic Brain Injury.

[d] Exact numbers are not displayed due to cell size suppression requirements

[e] Of beneficiaries who were full benefit dual-eligible in January, 2014 (n=206,874)

Abbreviations: HCBS = home and community-based services; BH = behavioral health; CAP= Community Alternatives Programs

© 2023 Kaufman B, et al, JAMA Health Forum

eTable 3. Dual status in January 2014 by need-based subgroup

Variable	Overall	Community well	HCBS	Nursing home resident	Intensive BH service user	1915(c) waiver	1915(b) waiver
N		213,667	50,095	24,927	50,509	17,215	4,277
Dual Enrollment Status in January 2014							
Dual eligible, full[a]	206,874 (62.1%)	120,958 (56.6%)	39,502 (78.9%)	13,676 (54.9%)	37,024 (73.3%)	14,823 (86.1%)	3,476 (81.3%)
Dual eligible, partial	13,785 (4.1%)	10,407 (4.9%)	1,560 (3.1%)	1,079 (4.3%)	882 (1.7%)	279 (1.6%)	15 (0.4%)
Enrolled in Medicaid, not Medicare	27,329 (8.2%)	18,515 (8.7%)	2,880 (5.7%)	254 (1.0%)	6,387 (12.6%)	1,286 (7.5%)	749 (17.5%)
Enrolled in Medicare, not Medicaid	54,377 (16.3%)	36,808 (17.2%)	5,103 (10.2%)	9,585 (38.5%)	3,581 (7.1%)	651 (3.8%)	21 (0.5%)
Not enrolled in Medicaid nor Medicare	30,875 (9.3%)	26,979 (12.6%)	1,050 (2.1%)	333 (1.3%)	2,635 (5.2%)	176 (1.0%)	16 (0.4%)
Changes in eligibility status among those who were full duals in January 2014							
Full dual status entire period or through death	147,738 (71.4%)	82,906 (68.5%)	29,939 (75.8%)	9,021 (66.0%)	30,075 (81.2%)	11,725 (79.1%)	3,203 (92.1%)
Any change to partial dual status	13,405 (6.5%)	9,976 (8.2%)	1,571 (4.0%)	269 (2.0%)	1,661 (4.5%)	622 (4.2%)	97 (2.8%)
Any loss of Medicaid enrollment	50,624 (24.5%)	31,931 (26.4%)	8,497 (21.5%)	4,486 (32.8%)	5,710 (15.4%)	2,619 (17.7%)	182 (5.2%)
Died during study period	47,240 (22.8%)	22,740 (18.8%)	12,117 (30.7%)	7,570 (55.4%)	4,191 (11.3%)	4,229 (28.5%)	209 (6.0%)

[a]This subgroup is evaluated for changes in enrollment status in the following eFigure 2

© 2023 Kaufman B, et al, JAMA Health Forum

eFigure 2. Changes in Dual Status throughout the Study Period by Need-Based Subgroup, among those who were full-benefit dual eligible (FBDE) beneficiaries in January 2014[a]

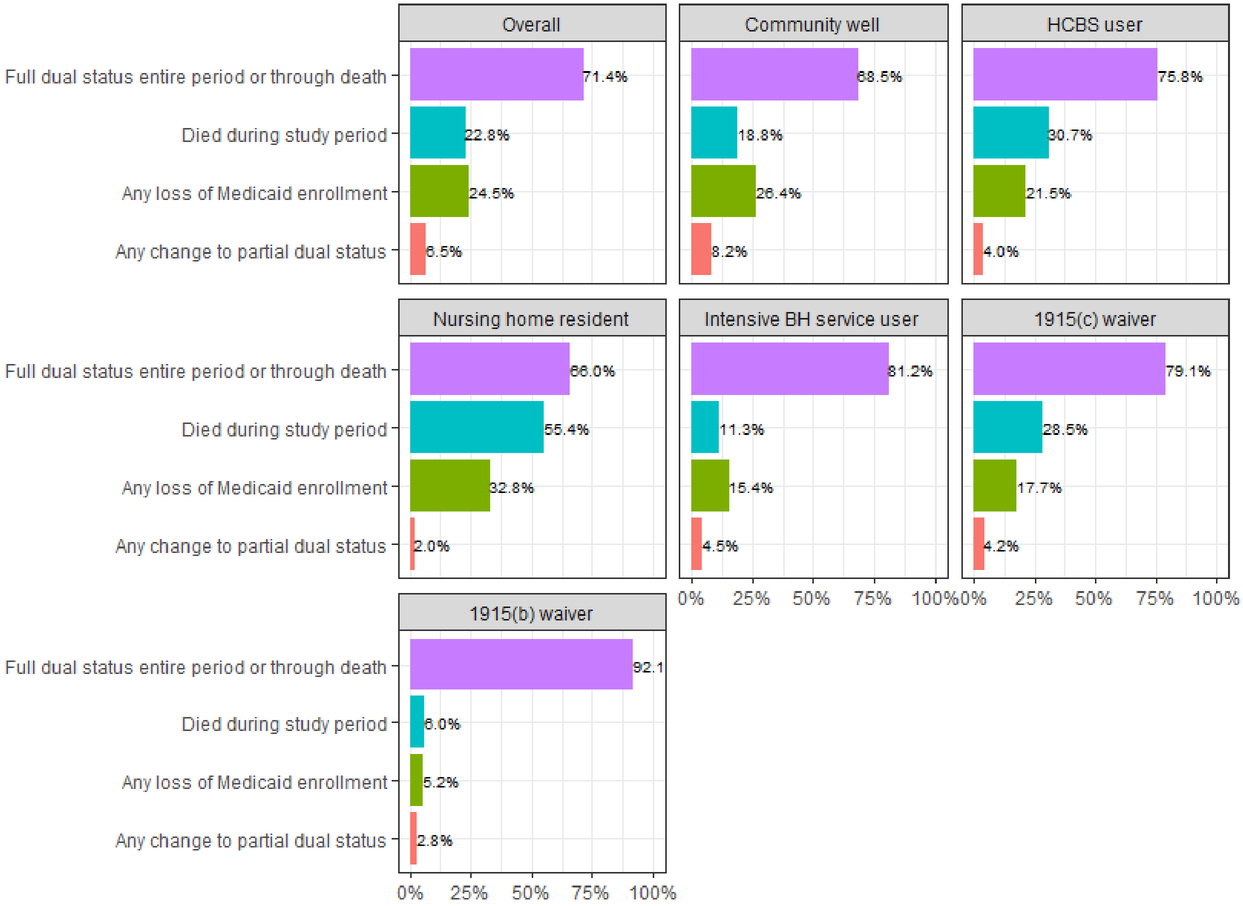

[a] FBDE beneficiaries in January 2014 are shown in the preceding eTable 3 (“Dual eligible, full” row) and are the denominator for the evaluation of changes over the study period. Subgroup enrollment was assessed as ever meeting the condition(s) for that subgroup, at any time during the study period. Enrollment in multiple subgroups does not necessarily indicate concurrent receipt of these services.

© 2023 Kaufman B, et al, JAMA Health Forum

eTable 4. Overlap in beneficiaries between need-based subgroups

Subgroup	Subgroups					
	**Community well[a] N= N (%)**	**HCBS N=50,095 N (%)**	**Nursing home resident N=24,927 N (%)**	**Intensive BH service user N=50,509 N (%)**	**1915(c) waiver N=17,215 N (%)**	**1915(b) waiver N=4,277 N (%)**
Community well		0 (%)	0 (%)	0 (%)	0 (%)	0 (%)
HCBS	0 (0%)		1,722 (6.9%)	10,736 (21.3%)	3,561 (20.7%)	157 (3.7%)
Nursing home resident	0 (0%)	1,722 (3.4%)		1,920 (3.8%)	428 (2.5%)	20 (0.5%)
Intensive BH service user	0 (%)	10,736 (21.4%)	1,920 (7.7%)		5,874 (34.1%)	4,220 (98.7%)
CAP waiver	0 (%)	3,561 (7.1%)	428 (1.7%)	5,874 (11.6%)		4,277 (100%)
Innovations waiver	0 (%)	157 (0.3%)	20 (0.1%)	4,220 (8.4%)	4,277 (24.8%)	

[a] Community well is defined as not enrolled in any need-based subgroup

Percentages are out of the column total. Subgroup enrollment was assessed as ever meeting the condition(s) for that subgroup at any time during the study period. Enrollment in multiple subgroups does not necessarily indicate they were receiving these services concurrently.
HCBS = home- and community-based services; BH = behavioral health services

© 2023 Kaufman B, et al, JAMA Health Forum

eTable 5. Proportion of total fee-for-service healthcare spending per person-year funded by Medicaid Programs among North Carolina FBDE beneficiaries, overall

Variable	Medicaid	Medicare	Combined	Proportion of Combined Costs Attributed to Medicaid
Overall Spending	$12,698.55	$14,175.06	$26,873.61	47.3%
Inpatient	$85.92	$5,231.20	$5,317.12	1.6%
Outpatient facility	$4,199.26	$2,938.77	$7,138.03	58.8%
Carrier	$3,093.58	$3,120.52	$6,214.10	49.8%
SNF[a]	NA	$1,340.67	$1,340.67	NA
Home Health	$183.55	$554.17	$737.72	24.9%
Hospice	$208.08	$551.98	$760.06	27.4%
DME	$34.65	$437.73	$472.38	7.3%
NH, SNF, and ICF[b,c]	$4,730.95	NA	$4,730.95	NA
Dental[b]	$162.53	NA	$162.53	NA

[a] Services covered by Medicare only
[b] Services covered by Medicaid only
[c] Institutional costs only for skilled nursing facilities (SNF), nursing homes (NH), and intermediate care facilities (ICF)

© 2023 Kaufman B, et al, JAMA Health Forum

eTable 6. Proportion of total fee-for-service healthcare spending per person-year funded by Medicaid Programs among Community Well beneficiaries

Variable	Medicaid	Medicare	Combined	Proportion of Combined Costs Attributed to Medicaid
Overall Spending	$7,604.51	$12,129.01	$19,733.52	38.5%
Inpatient	$66.29	$4,456.06	$4,522.35	1.5%
Outpatient facility	$3,957.01	$2,812.29	$6,769.30	58.5%
Carrier	$498.93	$2,783.12	$3,282.05	15.2%
SNF[a]	NA	$996.87	$996.87	NA
Home Health	$71.38	$288.51	$359.89	19.8%
Hospice	$199.80	$402.77	$602.57	33.2%
DME	$19.25	$389.38	$408.63	4.7%
NH, SNF, and ICF[b,c]	$2,627.78	NA	$2,627.78	NA
Dental[b]	$164.04	NA	$164.04	NA

[a] Services covered by Medicare only
[b] Services covered by Medicaid only
[c] Institutional costs only for skilled nursing facilities (SNF), nursing homes (NH), and intermediate care facilities (ICF)

© 2023 Kaufman B, et al, JAMA Health Forum

eTable 7. Proportion of total fee-for-service healthcare spending per person-year funded by Medicaid Programs among 1915(c) Waiver beneficiaries

Variable	Medicaid	Medicare	Combined	Proportion of Combined Costs Attributed to Medicaid
Overall Spending	$25,264.46	$18,441.55	$43,706.01	57.8%
Inpatient	$77.30	$6,873.61	$6,950.91	1.1%
Outpatient facility	$5,869.42	$3,003.12	$8,872.54	66.2%
Carrier	$16,065.65	$3,624.36	$19,690.01	81.6%
SNF[a]	NA	$1,702.04	$1,702.04	NA
Home Health	$903.40	$1,470.81	$2,374.21	38.1%
Hospice	$95.11	$811.01	$906.12	10.5%
DME	$170.44	$956.58	$1,127.02	15.1%
NH, SNF, and ICF[b,c]	$1,946.21	NA	$1,946.21	NA
Dental[b]	$136.90	NA	$136.90	NA

[a] Services covered by Medicare only
[b] Services covered by Medicaid only
[c] Institutional costs only for skilled nursing facilities (SNF), nursing homes (NH), and intermediate care facilities (ICF)

© 2023 Kaufman B, et al, JAMA Health Forum

eTable 8. Proportion of total fee-for-service healthcare spending per person-year funded by Medicaid Programs among Home- and Community-Based Service (HCBS) users

Variable	Medicaid	Medicare	Combined	Proportion of Combined Costs Attributed to Medicaid
Overall Spending	$19,106.99	$20,962.08	$40,069.07	47.7%
Inpatient	$101.02	$7,829.40	$7,930.42	1.3%
Outpatient facility	$5,494.00	$3,518.93	$9,012.93	61.0%
Carrier	$10,260.81	$4,262.27	$14,523.08	70.7%
SNF[a]	NA	$2,122.76	$2,122.76	NA
Home Health	$478.60	$1,612.64	$2,091.24	22.9%
Hospice	$127.68	$1,019.46	$1,147.14	11.1%
DME	$68.75	$596.60	$665.35	10.3%
NH, SNF, and ICF[b,c]	$2,435.55	NA	$2,435.55	NA
Dental[b]	$140.55	NA	$140.55	NA

[a] Services covered by Medicare only
[b] Services covered by Medicaid only
[c] Institutional costs only for skilled nursing facilities (SNF), nursing homes (NH), and intermediate care facilities (ICF)

© 2023 Kaufman B, et al, JAMA Health Forum

eTable 9. Proportion of total fee-for-service healthcare spending per person-year funded by Medicaid Programs among Intensive Behavioral Health service users

Variable	Medicaid	Medicare	Combined	Proportion of Combined Costs Attributed to Medicaid
Overall Spending	$12,340.18	$12,603.18	$24,943.36	49.5%
Inpatient	$169.10	$5,274.34	$5,443.44	3.1%
Outpatient facility	$4,105.60	$2,296.42	$6,402.02	64.1%
Carrier	$3,836.10	$3,108.58	$6,944.68	55.2%
SNF[a]	NA	$860.18	$860.18	NA
Home Health	$187.72	$479.32	$667.04	28.1%
Hospice	$78.36	$226.47	$304.83	25.7%
DME	$30.16	$357.83	$387.99	7.8%
NH, SNF, and ICF[b,c]	$3,734.61	NA	$3,734.61	NA
Dental[b]	$198.50	NA	$198.50	NA

[a] Services covered by Medicare only
[b] Services covered by Medicaid only
[c] Institutional costs only for skilled nursing facilities (SNF), nursing homes (NH), and intermediate care facilities (ICF)

© 2023 Kaufman B, et al, JAMA Health Forum

eTable 10. Proportion of total fee-for-service healthcare spending per person-year funded by Medicaid Programs among Nursing Home Residents

Variable	Medicaid	Medicare	Combined	Proportion of Combined Costs Attributed to Medicaid
Overall Spending	$47,950.75	$20,408.71	$68,359.46	70.1%
Inpatient	$103.88	$5,727.40	$5,831.28	1.8%
Outpatient facility	$1,998.86	$4,420.81	$6,419.67	31.1%
Carrier	$971.55	$3,650.46	$4,622.01	21.0%
SNF[a]	NA	$4,642.40	$4,642.40	NA
Home Health	$58.72	$256.12	$314.84	18.7%
Hospice	$1,006.64	$1,393.85	$2,400.49	41.9%
DME	$7.05	$317.63	$324.68	2.2%
NH, SNF, and ICF[b,c]	$43,685.54	NA	$43,685.54	NA
Dental[b]	$118.48	NA	$118.48	NA

[a] Services covered by Medicare only
[b] Services covered by Medicaid only
[c] Institutional costs only for skilled nursing facilities (SNF), nursing homes (NH), and intermediate care facilities (ICF)

© 2023 Kaufman B, et al, JAMA Health Forum
