# Supplementary material for: Health Care Use and Spending Among Need-Based Subgroups of Medicare Beneficiaries With Full Medicaid Benefits
Source: JAMA Health Forum. 2023 May 12;4(5):e230973. doi: 10.1001/jamahealthforum.2023.0973 (PMC10182424; doi:10.1001/jamahealthforum.2023.0973)
Supplement: Supplement 2. — Data Sharing Statement [file jamahealthforum-e230973-s002.pdf]

Data Sharing Statement

Kaufman. Health Care Use and Spending Among Need-Based Subgroups of Medicare Beneficiaries With Full Medicaid Benefits. *JAMA Health Forum*. Published May 12, 2023. doi:10.1001/jamahealthforum.2023.0973

Data
Data available: No

Additional Information
Explanation for why data not available: Data used for this study were available under institutional agreements with the Centers for Medicare and Medicaid Services and the North Carolina Department of Health and Human Services. Sharing of individual or raw data is prohibited under the terms of the Data Use Agreements.
